# miR-27a induced by colon cancer cells in HLECs promotes lymphangiogenesis by targeting SMAD4

**DOI:** 10.1371/journal.pone.0186718

**Published:** 2017-10-24

**Authors:** Qi Xu, Jin-Lu Tong, Chen-Peng Zhang, Qian Xiao, Xiao-Lin Lin, Xiu-Ying Xiao

**Affiliations:** 1 Department of Oncology, Renji Hospital, School of Medicine, Shanghai Jiao Tong University, Shanghai, China; 2 Division of Gastroenterology and Hepatology, Renji Hospital, Shanghai Institute of Digestive Disease, School of Medicine, Shanghai JiaoTong University, Shanghai, China; 3 Department of Nuclear Medicine, Renji Hospital, School of Medicine, Shanghai Jiaotong University, Shanghai, China; 4 Department of Pharmacology, Yale School of Medicine, New Haven, Connecticut, United States of America; Universitat de Barcelona, SPAIN

## Abstract

**Aim:**

Metastasis of tumor cells occurs through lymphatic vessels, blood vessels and transcoelomic spreading. Growing evidence from *in vivo* and *in vitro* studies has indicated that tumor lymphangiogenesis facilitates metastasis. However, the regulation of lymphangiogenesis in colon cancer remains unclear. The aims of this study were to identify key miRNAs in colon cancer lymphangiogenesis and to investigate its target and mechanism.

**Methods:**

miRNA microarray analysis was conducted to identify miRNAs in human lymphatic endothelial cells (HLECs) that were regulated by co-cultured human colon cancer cells. Gain- and loss-of-function studies were performed to determine the function of miR-27a, a top hint, on lymphangiogenesis and migration in HLECs. Furthermore, bioinformatics prediction and experimental validation were performed to identify miR-27a target genes in lymphangiogenesis.

**Results:**

We found that expression of miR-27a in HLECs was induced by co-culturing with colon cancer cells. Over-expression of miR-27a in HLECs enhanced lymphatic tube formation and migration, whereas inhibition of miR-27a reduced lymphatic tube formation and migration. Luciferase reporter assays showed that miR-27a directly targeted *SMAD4*, a pivotal component of the TGF-β pathway. In addition, gain-of-function and loss-of-function experiments showed that SMAD4 negatively regulated the length of lymphatic vessels formed by HLECs and migration.

**Conclusions:**

Our data indicated that colon cancer cell induced the expression of miR-27a in HLECs, which promoted lymphangiogenesis by targeting *SMAD4*. Our finding implicated miR-27a as a potential target for new anticancer therapies in colon cancer.

## Introduction

Colon cancer is the fourth most common cancer type and the second leading cause of cancer-related death worldwide [1]. Colon cancer is associated with significant mortality because it usually metastasizes and spreads throughout the body. Therapeutic options are limited for patients with metastatic colon cancer. Thus, it is critical to understand the mechanism of metastasis in colon cancer and develop novel therapeutic strategies. The lymphatic system plays key roles in cancer development and, specifically, in cancer metastasis [2]. The presence of tumor cells in regional or sentinel lymph nodes is a key predictor of outcome in colon cancer [3]. Dynamic changes of the lymphatic vessels in tumor tissues, including lymphangiogenesis, facilitate metastasis [4]. However, the molecular mechanism underlying tumor lymphangiogenesis has been less rigorously studied, compared to angiogenesis.

miRNAs are short non-coding RNAs that negatively regulate gene expression by inhibiting protein translation or accelerating mRNA degradation. miRNAs have important roles in many biological processes, including cell cycle regulation, cell growth, apoptosis, differentiation and the cellular response to stress [5]. The emergence of miRNAs as key players in the tumorigenesis, progression, and metastasis of cancer has led to new diagnostic and therapeutic opportunities [6]. In colon cancer, microRNA expression profiles were associated with prognosis and therapeutic outcome [7]. Some miRNAs were found to be oncogenic in colon cancer, including the miR-17/92 cluster [8] and miR-21 [9]. On the other hand, some miRNAs were found to be tumor suppressors in colon cancer, including let-7 [10], miR-143 [11], and miR-145 [9]. Although miRNAs function as key modulators of angiogenesis [12], the critical roles of miRNAs in colon cancer lymphangiogenesis remain unclear. In this study, we have identified miR-27a as a key regulator of lymphangiogenesis by targeting *SMAD4* in colon cancer.

## Materials and methods

### Cell culture and tumor-HLEC co-culture

The human colon cancer cell lines SW620 and SW480 were obtained from the American Type Culture Collection (ATCC), cultured in Dulbecco's Modified Eagle Medium (Hyclone laboratories, South Logan, UT, USA) supplemented with 10% fetal bovine serum (FBS) (Invitrogen Life Technologies, Carlsbad, CA, USA), 100 U/mL penicillin (Invitrogen Life Technologies, Carlsbad, CA, USA), and 100 μg/mL streptomycin (Invitrogen Life Technologies, Carlsbad, CA, USA) at 37°C in a humidified atmosphere of 5% CO_2_.

Human lymphatic endothelial cells (HLECs) were obtained from ScienCell Research Laboratories (ScienCell, San Diego, CA, USA) and maintained in Endothelial cell medium (ECM) (ScienCell, San Diego, CA, USA) supplemented with 10% FBS, 100 U/mL penicillin, and 100 μg/mL streptomycin at 37°C in a humidified atmosphere of 5% CO_2_. Prior to assays, HLECs were incubated overnight with 10 ml of sterile Dulbecco’s phosphate buffered saline (DPBS) and 150 μl (1 mg/ml) of fibronectin stock solution.

For the tumor cell-HLECs co-culture system, human colon cancer cells were plated in 35-mm dishes. HLECs were then seeded on cell-culture inserts containing a polycarbonate membrane with a 0.4-μm pore (Millicell, Millipore, Billerica, MA, USA) placed these dishes and incubate for 48 hours.

### RNA isolation and miRNA microarray analysis

Total RNA, including miRNA, was isolated using Trizol reagent (Invitrogen Life Technologies, Carlsbad, CA, USA) according to the manufacturer’s instructions from HLECs co-cultured with colon cancer cell lines.

The isolated miRNAs were then labeled with Hy3TM using the miRCURYTM Array Labelling kit (Exiqon, Vedbaek, Denmark) and then hybridized on miRCURYTM LNA microRNA Array 16.0 edition (Exiqon, Vedbaek, Denmark), as previously described [13]. Hybridization images were collected using a GenePix 4000B laser scanner (Molecular Devices, Sunnyvale, CA, USA). Images were quantified using GenePix Pro 6.0 (Axon Instruments, Sunnyvale, CA, USA). Raw data were further processed in Microsoft Excel.

### Real-time qRT-PCR

cDNAs were generated using a reverse transcription kit (Fermentas, Glen Burnie, MD, USA) according to the manufacturer’s instructions. Real-time quantitative PCR experiments were performed with SYBR Green PCR Master Mix (Takara, Dalian, China) and on an ABI 7900 sequence detection system (Applied Biosystems, San Diego, CA, USA), according to the manufacturer’s protocol. The primers are listed as follows: *SMAD4* sense, 5-AGTAACGATGCCTGTCTGA-3, antisense, 5-TGAAGTCGTCCATCCAAT-3; *PROX-1* sense, 5-CAGCCCGAAAAGAACAGAAG-3, antisense, 5-GGGTCTAGCTCGCACATCTC-3; *GAPDH* sense, 5-GCACCGTCAAGGCTGAGAAC-3, antisense, 5-ATGGTGGTGAAGACGCCAGT-3. The TaqMan MicroRNA Reverse Transcription kit and TaqMan MicroRNA Assays were used to detect and quantify mature hsa-miR-27a-3p, hsa-miR-146a-5p, hsa-miR-20b-3p and hsa-miR-519e-5p. miRNA expression levels were normalized according to the expression of *RNU6B*. All primers were provided by Applied Biosystems. Experiments were performed in triplicate.

### Western blot

Total protein was extracted with RIPA buffer supplemented with protease inhibitors. Protein extracts were resolved on 10% SDS–PAGE gels, transferred onto PVDF membranes (Millipore, Billerica, MA, USA), as descripted previously [14]. The primary antibodies anti-rabbit Smad4 (Cell Signaling, Danvers, MA, USA) or GAPDH (Kang Cheng Biotechnology, Shanghai, China) were applied overnight. The dilutions of the primary antibodies were 1:1000 for anti-SMAD4 and 1:10000 for anti-GAPDH. The following day, the membranes were incubated with HRP-conjugated anti-rabbit secondary antibodies (Kang Cheng Biotechnology, Shanghai, China) at room temperature for 60 minutes. A chemiluminescence detection system (Millipore, Billerica, MA, USA) was used for visualization of the results.

### Transfection

For transfection studies, HLECs were plated at 50,000 cells per well 24 hours before transfection. Lipofectamine™ 2000 transfection reagent (Invitrogen Life Technologies, Carlsbad, CA, USA) were used to transfect plasmid, siRNA, miR-27a mimic, or miR-27a inhibitor, according to the manufacturer’s instructions. Expression plasmids for Smad4, namely pRK5-FLAG-Smad4, were kindly provided by Dr. Bert Vogelstein. Scrambled oligonucleotide (Genepharm, Shanghai, China), Smad4 siRNA (Genepharm, Shanghai, China), miR-27a mimic (Genepharm, Shanghai, China) and miR-27a inhibitor (Genepharm, Shanghai, China) were transfected into HLECs at the indicated concentrations.

### Tube formation assay

Matrigel (BD Biosciences, San Jose, CA, USA) was mixed with ECM at a 1:2 ratio. 50 ul of the mixture was added to each well in 96-well plates. After incubation at 37°C for 20 min for Matrigel solidification, pre-transfected HLECs were seeded on the 96-well plates and incubated in a 5% CO_2_ atmosphere at 37°C for 6 or 10 h. Pictures of tube formation were taken under a microscope, and the total lengths were quantified using Image J software.

### Cell migration assay

For cell migration analysis, 24-well chambers with 8-μm-pore polycarbonate membranes (Millipore, Billerica, MA, USA) were used. HLECs transfected with different concentrations of miR-27a mimic or inhibitor were trypsinized, suspended in ECM with no FBS, and then 1×10^5^ cells were seeded in the upper wells. ECM with 10% FBS was placed in the lower wells. After 8 or 12 h, cells that migrated to the bottom surface of the membrane were fixed with 100% methanol for 5 min, stained with 0.1% crystal violet for 10 min and counted in six random microscopic fields (200×) as previously described [15].

### Reporter constructs and luciferase activity assay

The 3’-UTR of the *SMAD4* gene was cloned into the 3’UTR of the OmicsLink^TM^ luciferase reporter vector (GeneCopoeia, Rockville, MD, USA). Mutagenesis was performed to generate reporter plasmids with mutations on miR-27a binding sites, as described in the reference [16]. HLECs were co-transfected with scrambled oligonucleotide, miR-27a mimic or inhibitor and OmicsLink^TM^ luciferase reporter vectors using Lipofectamine™ 2000. Twenty-four hours after transfection, luciferase activity was assayed with the Luc-Pair™ miR Luciferase Assay Kit (GeneCopoeia, Rockville, MD, USA) and a Promega Turner TD-20/20 Luminometer.

The plasmid P3TP-Lux was used to study the influence of miR-27a on the TGF-β signaling pathway and was kindly provided by Dr. Joan Massague (Memorial Sloan-Kettering Cancer Center, New York, NY, USA). HLECs were co-transfected with P3TP-Lux (1μg), pRL-TK (0.1μg), and different concentrations of miR-27a mimic, scrambled oligonucleotide or miR-27a inhibitor using Lipofectamine 2000. Twenty-four hours after the transfection, exogenous TGF-β1 (5 ng/ml) was added, and the luciferase assay was performed to measure the activity of firefly luciferase. Renilla luciferase activity was used for normalization.

### Data analysis and statistics

An unpaired t-test was used to assess the statistical significance of differences between groups. Pearson’s correlation coefficient was used to assess the association between miR-27a expression and *SMAD4* expression. All statistical analyses were performed using SPSS software 11.0 (Chicago, IL, USA) or GraphPad Prism 6 software (La Jolla, CA, USA). Data are presented as the mean ± SD. P<0.05 was considered statistically significant.

## Results

### microRNA profiling in HLECs co-cultured with colon cancer cells

**Fig 1:**
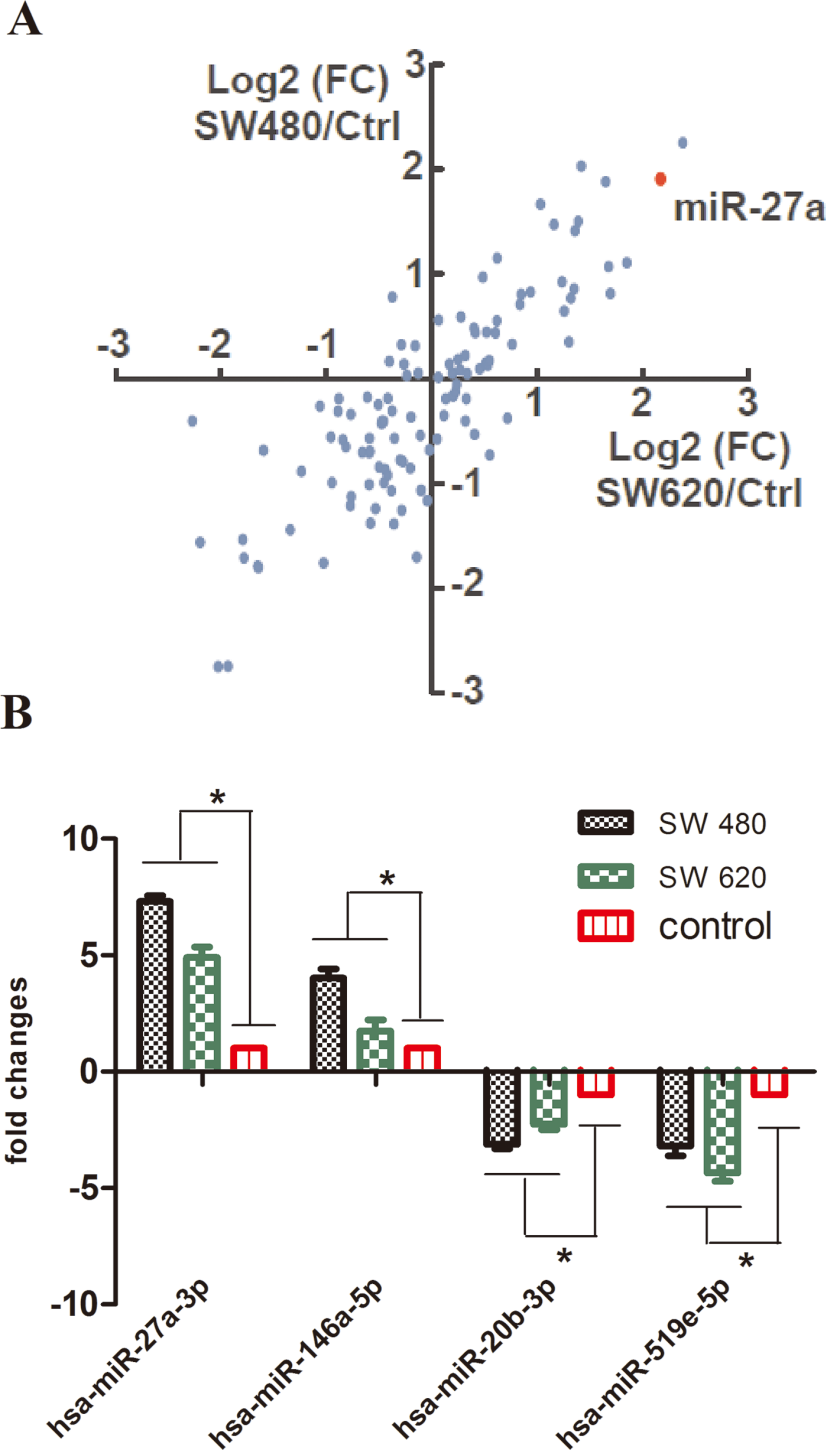
Differential miRNA expression in HLECs co-cultured with colon cancer cells. (A) The expression of miRNAs in co-cultured HLECs relative to their expression in HLECs alone. Unexpressed miRNAs were removed. (B) Real-time qRT-PCR analysis of four selected miRNAs in co-cultured HLECs compared with their expression in HLECs alone. Bars, means ± SD of 3 independent experiments. * P < 0.*05* versus miRNA expression in HLECs alone.

To identify key microRNAs in tumor-induced lymphangiogenesis, we performed co-cultures of HLECs with the human colon cancer cell lines, SW480 and SW620. After 48 h of co-culture, miRNA microarray analysis was used to compare the miRNA expression profiles in HLECs cultured alone versus HLECs co-cultured with different human colon cancer cell lines. The microarray analysis revealed 26 significantly downregulated miRNAs and 21 significantly upregulated miRNAs in HLECs co-cultured with human colon cancer cell lines compared to HLECs alone (Fig 1A). The top differentially expressed miRNAs were chosen for further studies. We then applied real-time qRT-PCR to validate the expression of two up-regulated miRNAs (hsa-miR-27a-3p and hsa-miR-146a-5p) and two down-regulated miRNAs (hsa-miR-20b-3p and hsa-miR-519e-5p) from the HLEC and colon cancer cell co-culture system. As shown in Fig 1B, the results of the qRT-PCR expression analysis of these four miRNAs were consistent with the results obtained from the miRNA array analysis. Because of the obvious up-regulation of miR-27a and because its function has been related to angiogenesis [17–18], we first chose miR-27a for further study.

### miR-27a promotes lymphangiogenesis and migration of HLECs

**Fig 2:**
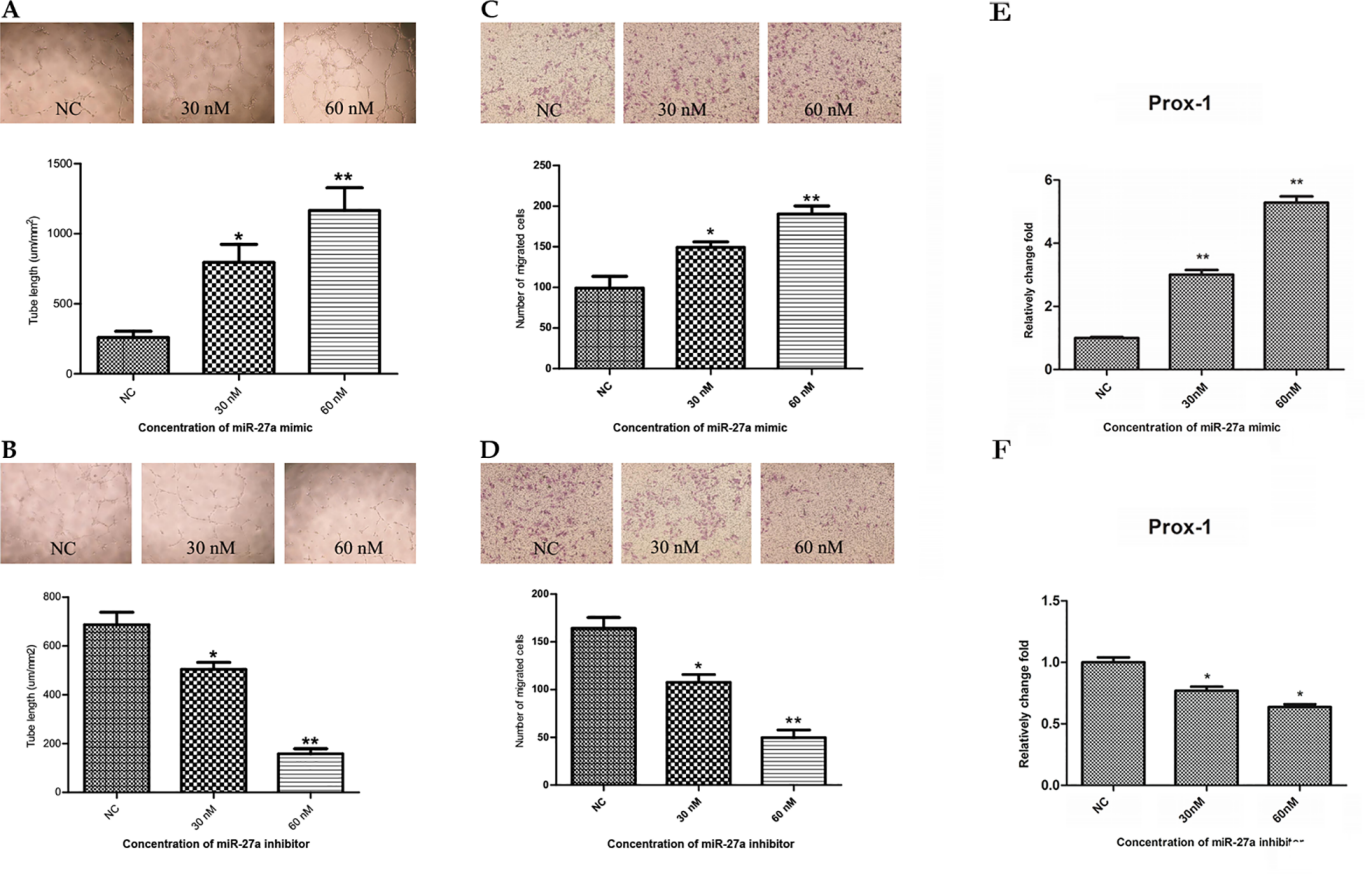
miR-27a increases tube formation of HLECs on Matrigel and promotes migration of HLECs. (A and B) Representative phase-contrast photographs and quantification of capillary-like tube formation in control or miR-27a mimic transfected HLECs (A) and control or miR-27a inhibitor transfected HLECs (B). Tube formation was quantified by the length of tubes. (C and D) Representative phase-contrast photographs and quantification of Transwell cell migration of control or miR-27a mimic transfected HLECs (C) and control or miR-27a inhibitor transfected HLECs (D). (E and F) Real-time qPCR analysis of Prox-1 expression in miR-27a mimic transfected HLECs or control (E) and miR-27a inhibitor transfected HLECs or control (F). Bars, means ± SD from 3 independent experiments. *P < 0.*05*, **P < 0.*01*, versus expression in negative control.

Cultured HLECs can form capillary-like tubes. Thus, they are widely used to mimic lymphangiogenesis *in vitro*. To assess the potential role of miR-27a in lymphangiogenesis, we treated HLECs with a miR-27a mimic and then examined lymphatic tube formation. We found that the lengths of the capillary-like tubes in Matrigel transfected with 30 nM or 60 nM miR-27a mimic for six hours were 3.07±0.91 fold (*P < 0*.*001*) and 4.49±0.63 fold (*P < 0*.*001*) longer than those observed in the control group, respectively (Fig 2A). We then transduced an miR-27a inhibitor into HLECs and monitored lymphatic tube formation and migration. In contrast to miR-27a mimic treated cells, the lengths of the capillary-like tubes in the cells transfected with the miR-27a inhibitor were 26.71±1.30% (*P < 0*.*05*) and 76.98±3.37% (*P < 0*.*001*) shorter than those observed in the control group after 10 hours of treatment with 30 nM or 60 nM miR-27a inhibitor, respectively (Fig 2B).

Transwell migration assays were then conducted to determine the function of miR-27a on the migration in HLECs. We found that the number of migrated cells increased after transduction with the miR-27a mimic in a dose-dependent manner. miR-27a mimic transfection at 30 nM or 60 nM enhanced migration by 1.50±0.28 fold (*P < 0*.*05*) and 1.92±0.18 fold (*P < 0*.*001*), respectively (Fig 2C). On the other hand, the number of migrating cells in HLECs transfected with 30 nM or 60 nM miR-27a mimic were 34.48±4.60% (*P < 0*.*05*) and 69.78±4.28% (*P < 0*.*001*) lesser than the control group, respectively (Fig 2D).

Prox-1 is required for lymphatic endothelial cell differentiation and promote lymphangiogenesis in vivo and in vitro [19]. It was one of the indications of lymphatic vasculature formation start [20]. We chose Prox-1 as biomarker to show the lymphangiogenesis modulated by miR-27a in HLECs. Quantitative PCR (qPCR) was conducted to determine Prox-1 expression in HLECs after transfected with miR-27a mimic or inhibitor. The results showed that Prox-1 expression increased compared with control by 3.00±0.26 fold (*P < 0*.*001*) and 5.28±0.36 fold (*P < 0*.*001*) after 30nM and 60nM miR-27a mimic transfection, respectively (Fig 2E). And conversely, Prox-1 expression level was 25.00±3.90% (*P < 0*.*05*) and 36.00±2.60% (*P < 0*.*05*) decreased after transfected with miR-27a inhibitor in 30nM and 60nM concentration, respectively (Fig 2F). The results consistent with the HLECs lymphatic tube formation and cell migration

### *SMAD4* is a target of miR27 in HLECs

**Fig 3:**
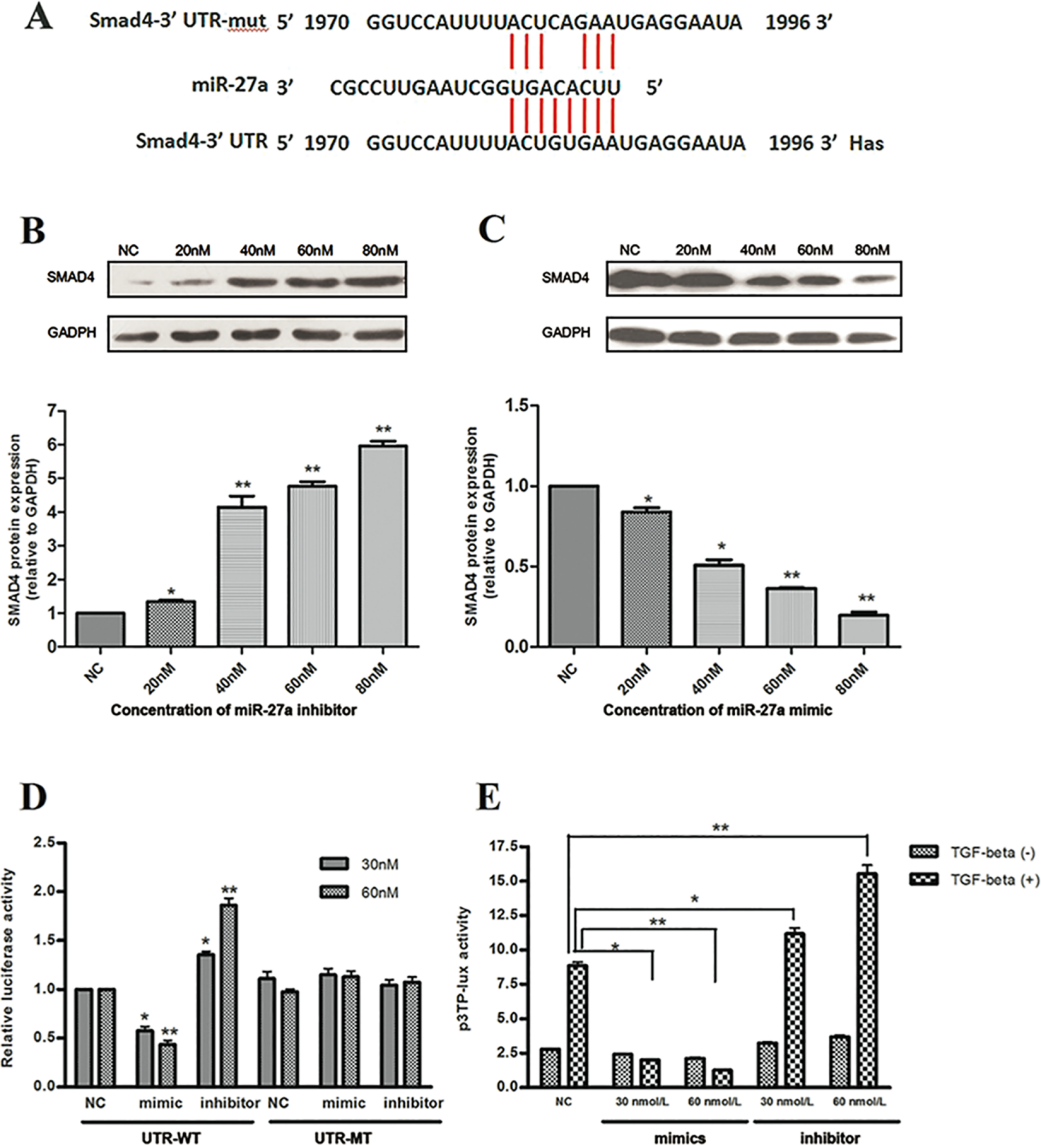
miR-27a directly targets SMAD4. (A) The predicted target site of has-miR-27a (middle) in the SMAD4-3’UTR region (bottom) as detected by TargetScan. A mutant SMAD4-3’UTR sequence was also shown (upper). (B) The quantification of SMAD4 protein expression in HLECs transfected with different concentrations of miR-27a inhibitor. (C) The quantification of SMAD4 protein expression in HLECs transfected with different concentrations miR-27a mimic. (D) The activity of wildtype or mutant SMAD4-3’UTR in HLECs co-transfected with control, miR-27a mimic, or miR-27a inhibitor in luciferase reporter assays. Relative luciferase activity was normalized to the scrambled oligonucleotide control. (E) The induction of the TGF-β1 pathway in HLECs transfected with control, miR-27a mimic, or miR-27a inhibitor in luciferase reporter assays. Relative luciferase activity was normalized to the scrambled oligonucleotide control. *P < 0.*05*, **P < 0.*01*.

To identify possible miR-27a targets in lymphangiogenesis, we conducted microRNA target prediction with widely used tools, including DIANA, TargetScan, and PITA. Among the predicted miR-27a targets, the *SMAD4* gene (Fig 3A) was chosen for further analysis as all three prediction methods listed *SMAD4* as a top candidate and miR-27a has an established role in the inhibition of tumor metastasis. The bioinformatics analysis suggested that miR-27a binds to the *SMAD4* mRNA 3’UTR and down-regulates the synthesis of SMAD4 protein to potentially regulate lymphangiogenesis.

To investigate the relationship between miR-27a and *SMAD4*, Western blot was used to detect SMAD4 protein expression in HLECs transfected with different concentrations of miR-27a mimic or miR-27a inhibitor. Compared with scrambled oligonucleotide, SMAD4 protein expression was decreased in miR-27a mimic transfected HLECs and increased in miR-27a inhibitor transfected HLECs in a concentration-dependent manner (Fig 3B and 3C).

To determine if *SMAD4* is a direct target of miR-27a, we cloned the 3’UTR of *SMAD4* mRNA into a luciferase reporter vector. Point mutants were also introduced into the construct to disrupt the predicted miR-27a binding site (Fig 3A). We co-transfected wild-type or mutant *SMAD4* 3’UTR luciferase reporter plasmids and miR-27a mimic or miR-27a inhibitor into HLECs. The luciferase activity was assayed after 24 hours of transfection. We found that the luciferase activity of wild-type *SMAD4* 3’-UTR reporter transfected cells was significantly suppressed when co-transfected with 30 nM and 60 nM miR-27a mimic (a decrease of 42.33±7.02% and 56.67±7.77%, respectively, *P < 0*.*001*), while the luciferase activity was significantly elevated following transfection with 30 nM and 60 nM miR-27a inhibitor (an increase of 35.33±6.03% and 86.33±12.22%, respectively, *P < 0*.*001*). In contrast, no significant difference was observed when the mutant SMAD4 3’UTR plasmid was used (Fig 3D). These results suggested that miR-27a directly regulates *SMAD4* by binding to the 3’UTR of *SMAD4* mRNA.

### The miR-27a and TGF-β signaling pathway in lymphangiogenesis

SMAD4 is a key component of the TGF-β signaling pathway, which negatively regulates lymphangiogenesis [21]. We hypothesized that miR-27a regulates the TGF-β signaling pathway in HLECs. To test this hypothesis, we used the dual-luciferase reporter plasmid p3TP-Lux. In this plasmid, a luciferase reporter is transcribed from the promoter of the plasminogen activator inhibitor-1 (*PAI-1*) gene, a TGF-β induced promoter. We co-transduced p3TP-Lux and miR-27a mimic or miR-27a inhibitor into HLECs, treated cells with exogenous TGF-β1, and then determined luciferase activity. Compared with scrambled oligonucleotide, miR-27a mimic significantly suppressed the TGF-β1 response (decreased 77.40±1.88% and 85.79±1.93% for the 30 nM and 60 nM miR-27a mimic, respectively, *P = 0*.*02* and *P < 0*.*01*). On the other hand, the TGF-β1 induced response was significantly elevated in HLECs transfected with miR-27a inhibitor (increased 26.00±0.02% and 75.00±0.07% when treated with 30 nM and 60 nM miR-27a inhibitor, respectively, *P = 0*.*03* and *P < 0*.*01*). However, miR-27a showed no significant effects on the basal level of *PAI-1* promoter activity without exogenous addition of TGF-β1 (*P > 0*.*05*) (Fig 3E). This result suggested that miR-27a negatively regulates the Smad4-TGF-β signaling pathway in HLECs.

### SMAD4 inhibits lymphangiogenesis and migration of HLECs

**Fig 4:**
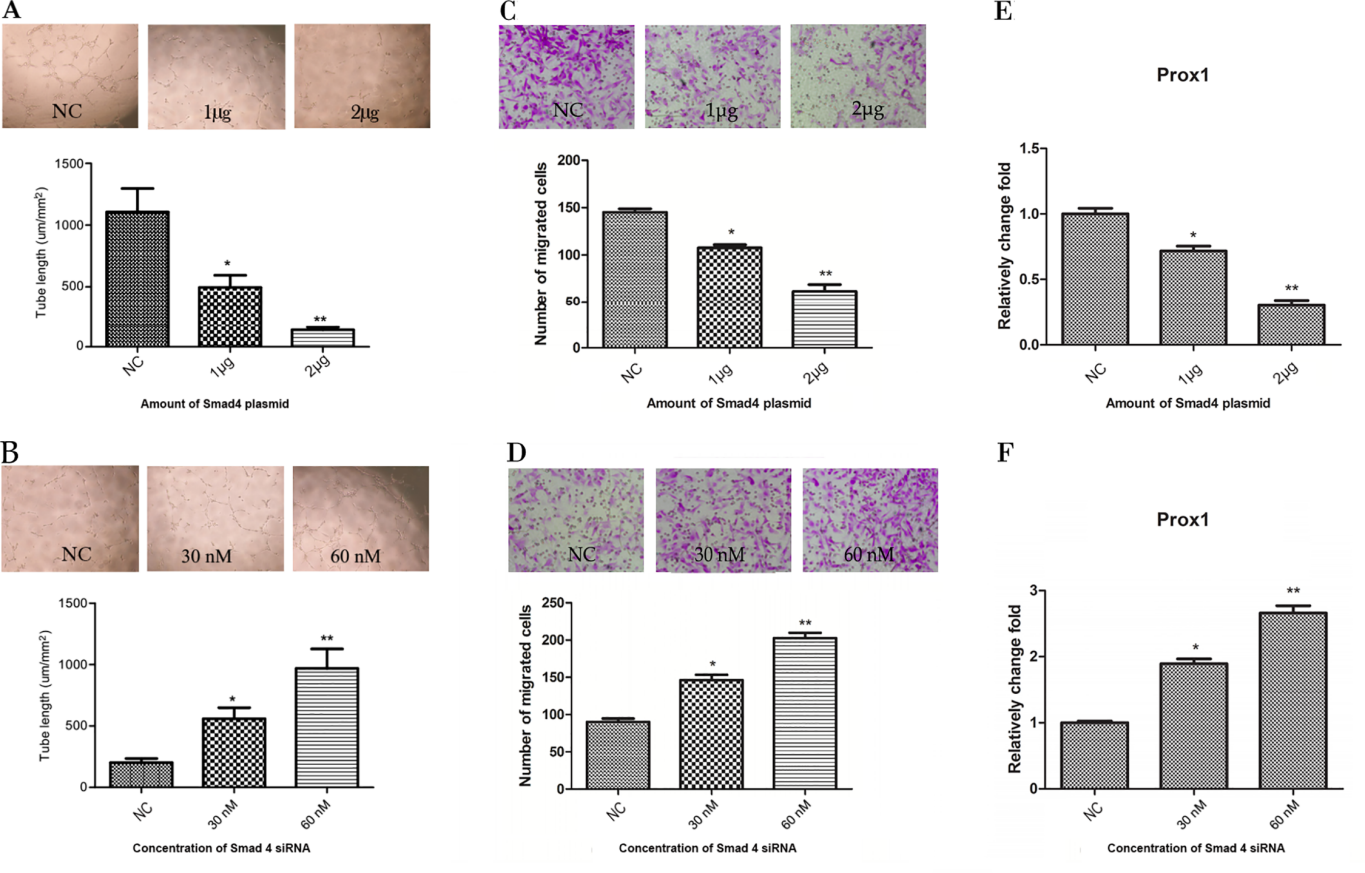
*SMAD4* alters the tube formation of HLECs. (A and B) Representative phase-contrast photographs and quantification of capillary-like tube formation in control or SMAD4 overexpressed HLECs (A) and control or SMAD4 knockdown HLECs (B). Tube formation was quantified by the analyzing the length of tubes. (C and D) Representative phase-contrast photographs and quantification of Transwell cell migration of control or SMAD4 over-expression plasmid transfected HLECs or control (C) and SMAD4 siRNA or negative control transfected HLECs (D). (E and F) Relatively fold change of Prox-1 in SMAD4 over-expression plasmid transfected HLECs and empty vector control (E) or SMAD4 siRNA transfected HLECs and control (F). Bars, means ± SD from 3 independent experiments. *P < 0.*05*, **P < 0.*01*, versus negative control.

The role of SMAD4 in lymphangiogenesis is not fully understood. To further investigate the effect of *SMAD4* in colon cancer lymphangiogenesis, we overexpressed or knocked down SMAD4 in HLECs and monitored tube formation. Compared with the empty vector control, the tube lengths decreased by 65.19 ± 1.22% with 1μg of the SMAD4 over-expression plasmid (*P < 0*.*05*), and 87.56 ± 3.90% after transfection with 2μg of the SMAD4 plasmid (*P < 0*.*001*) (Fig 4A). Conversely, compared with scrambled oligonucleotide, the tube lengths were increased 2.76±0.03 fold (*P < 0*.*05*) and 4.79±1.51 fold (*P < 0*.*001*) when HLECs were transfected 30 nM and 60 nM *SMAD4* siRNA, respectively (Fig 4B). The function of Smad4 on the HLECs migration was detected by transwell migration assay. It showed that the number of migrated cells decreased after transduction with SMAD4 over-expression plasmid in a dose-dependent manner. Migrated HLECs transfected with 1 μg or 2 μg SMAD4 over-expression plasmid were 25.79±4.93% (*P < 0*.*05*) and 57.38±2.75% (*P < 0*.*001*) less than empty vector control, respectively (Fig 4C). And on the other hand, migrated cells in HLECs after transfected with 30nM or 60nM SMAD4 siRNA were increased in 1.64±0.21 fold (*P < 0*.*05*) and 2.24±0.28 fold (*P < 0*.*001*) compared with scrambled oligonucleotide, respectively (Fig 4D). These results demonstrated that SMAD4 negatively regulated tube formation and migration of HLECs, which may inhibit tumor lymphangiogenesis *in vivo*.

Prox-1 expression level was also detected in the HLECs after transfected with SMAD4 over-expression plasmid and SMAD4 siRNA. Prox-1 expression was 28.33±1.76% (*P < 0*.*05*) and 69.00±3.74% (*P < 0*.*001*) decreased in the 1 μg or 2 μg SMAD4 over-expression plasmid transfected HLECs compared with empty vector control, respectively (Fig 4E). In contrast, HLECs with 30nM and 60nM SMAD4 siRNA transfection showed up-regulated Prox-1 expression in 1.89±0.21 fold (*P < 0*.*05*) and 2.66±0.19 fold (*P < 0*.*001*) compared with scrambled oligonucleotide control, respectively (Fig 4F).

## Discussion

Distant metastasis is the main cause of death for patients with colon cancer. Malignant cell invasion and metastasis are among the most important hallmarks of cancer and key targets in cancer therapy [22]. Lymphangiogenesis has traditionally been overshadowed by the greater emphasis placed on the blood vascular system (angiogenesis). Restricting lymphatic vessel growth may prevent lymph node metastasis, which is a crucial step during colon cancer progression. Tumor lymphangiogenesis molecular mechanism is complicated, and numerous factors such as Vascular Endothelial Growth Factors (VEGFs), platelet-derived growth factors (PDGFs), fibroblast growth factors (FGFs), and angiopoietins are proved to be important factors in this process. Among them, the VEGF-C/VEGF-D/VEGFR-3 signaling pathway is especially associated with lymphangiogenesis in cancer [23]. Considering the multiple pathways involved in lymphangiogenesis, abundant mechanisms may contribute to the formation of lymphatic vessels. Thus, effective anti-lymphangiogenic therapy should include multiple inhibitors targeting several components of the lymphangiogenesis process.

Studies have shown that miRNAs participate in multiple signaling pathways involved in angiogenesis and lympangiogenesis in different cancers. miR-126 was reported to play an important role in angiogenesis through inhibition of the RAS, ERK and PI3K/AKT signaling pathways and by regulating VEGFR-2-related signal transduction [24–26]. In addition, miR-126 was associated with angiogenesis and lymphangiogenesis in oral squamous cell carcinoma through VEGF-A activation [27]. miR-1236 was a negative regulator of VEGFR-3 in lymphangiogenesis [28] and miR-181 was found to be an important regulator of angiogenesis and lymphangiogenesis via Prox-1 inhibition [29]. Nevertheless, the precise mechanism underlying lymphangiogenesis remains under active investigation.

In this study, we examined the miRNA expression profiles of HLECs co-cultured with or without colon cancer cell lines. We identified multiple differentially expressed miRNAs. These results suggested that human colon cancer cells induced changes in the miRNA expression pattern of HLECs and that some of the miRNAs involved in this response might regulate lymphangiogenesis in human colon cancer.

Cancer cells secret factors to induce formation of new blood and lymph vessels in tumor tissues to satisfy the requirement for oxygen and nutrition [30]. One of the most famous examples is VEGFs [23, 31]. Secreted VEGFs bind to their receptors (VEGFRs) in endothelial cells, active multiple signal pathways, induce gene expression, regulate vascular permeability, and promote cell proliferation and migration [23, 31]. Interestingly, VEGF-C, which binds to VEGFR-3 on the lymphatic endothelial cells showed specific association with tumor lymphangiogenesis. If colon cancer cells induced miR-27a expression in HLECs by secreting VEGF-C or other VEGFs requires further studies.

Among all of the differentially expressed miRNAs, miR-27a was of the most interest. miR-27a is involved in the regulation of cancer cell proliferation, tumorigenesis, multidrug resistance and metastasis in various types of tumors [32–34]. miR-27a is considered an “onco-miRNA” that is possibly associated with tumor progression and metastasis in various cancers, including breast cancer, pancreatic cancer, and ovarian cancer [35–37]. Tang et al. [38] reported that miR-27a promotes angiogenesis by mediating the endothelial differentiation of BCSLCs. Therefore, we focused on the role of miR-27a in colon cancer lymphangiogenesis. We found that over-expression of miR-27a promoted lymphatic tube formation and HLEC migration. Conversely, down-regulation of miR-27a was associated with a decrease in lymphatic tube formation and HLEC migration. These results suggested that miR-27a was one of the key regulatory factors involved in HLEC lymphangiogenesis. Over the past decade, the value of miRNAs as therapeutic targets has been recognized. Some of these miRNA antagonists or inhibitors have been validated in clinically relevant animal models and have shown satisfactory efficacy in pre-clinical settings [39–40]. Here, the identification of the “onco-miRNA” miR-27a in lymphangiogenesis provides a new therapeutic opportunity for human colon cancer. *SMAD4* was a target of miR-27a in HLECs. First, bioinformatics analysis predicted SMAD4 carries a miR-27a target sequence on its 3’-UTR. Second, overexpression of an miR-27a mimic and an miR-27a inhibitor down- and up-regulated SMAD4 protein levels, respectively. Third, we showed that the *SMAD4* 3’UTR was targeted by miR-27a via dual-luciferase assays. Fourth, as revealed by the luciferase reporter assays, mutations on the predicted miR-27a binding site in the SMAD4 3’UTR inhibited the regulation. In addition, we showed that miR-27a negatively regulated the TGF-β pathway using a reporter system. These results suggested a miR-27a/SMAD4/TGF-β axis in HLECs that presumably regulates lymphangiogenesis.

*SMAD4* (DPC4) is a member of the SMAD family and is located on chromosome 18 (18q21) [41]. SMAD proteins are involved in the transcriptional regulation of the TGF-β signaling pathway. SMAD4 is a pivotal member of the TGF-β signaling pathway and functions as a tumor suppressor. Recent studies have shown that miRNAs are involved in the regulation of TGF-β/Smad signaling tumor suppression [42, 43]. Furthermore, some studies have shown a strong correlation between the high frequency of *SMAD4* gene mutations and colon cancer distant metastasis [44, 45]. The deletion of the *SMAD4* gene may lead to tumor angiogenesis and thus increase the metastatic potential of tumor cells [46]. Evidence has shown that SMAD4 can inhibit the expression of VEGF-C and, therefore, inhibit lymphangiogenesis in colon cancer [47]. These results are in agreement with the results described in this study. In our study, we showed that *SMAD4*, as a target of miR-27a, inhibited HLEC lymphatic tube formation.

Our results and others suggested pro-tumor function of miR-27 and anti-tumor function of SMAD4 in colon cancer. However, miR-27a and SMAD4 have also shown anti-cancer and oncogenic roles, respectively. For example, the expression of miR-27a was significantly reduced in prostate cancer. miR-27a inhibited prostate cancer cell proliferation and migration [48]. Overexpression of SMAD4 in hepatocellular carcinoma was associated with poor prognosis [49]. The functions of miR-27a and SMAD4 are likely tumor type- and cell type-dependent. They may also be regulated by other genetic or epigenetic alterations in tumor cells. Our study focused on the role of miR-27a and SMAD4 in HLECs and they may have different functions in tumor cells and endothelial cells.

## Conclusion

In summary, we demonstrated for the first time that miR-27a was a key regulator of lymphangiogenesis in human colon cancer and that it functioned via the TGF-β-SMAD4 signaling pathway. These findings indicated key roles of miR-27a in tumorigenesis and metastasis in human colon cancer and implicated miR-27a as a potential target for the development of new anticancer therapies.

## References

[1] Siegel RL, Miller KD, Jemal A. Cancer statistics, 2016. CA: a cancer journal for clinicians. 2016; 66(1):7–30. doi: 10.3322/caac.21332 . 26742998 10.3322/caac.21332

[2] Stacker SA, Achen MG, Jussila L, Baldwin ME, Alitalo K. Lymphangiogenesis and cancer metastasis. Nat Rev Cancer. 2002; 2(8):573–583. doi: 10.1038/nrc863 . 12154350 10.1038/nrc863

[3] Liang P, Hong JW, Ubukata H, Liu G, Katano M, Motohashi G, et al Myofibroblasts correlate with lymphatic microvessel density and lymph node metastasis in early-stage invasive colorectal carcinoma. Anticancer Res. 2005; 25(4):2705–2712. . 16080515

[4] Stacker SA, Williams SP, Karnezis T, Shayan R, Fox SB, Achen MG. Lymphangiogenesis and lymphatic vessel remodelling in cancer. Nat Rev Cancer. 2014;14(3):159–172. doi: 10.1038/nrc3677 . 24561443 10.1038/nrc3677

[5] Bartel DP. MicroRNAs: genomics, biogenesis, mechanism, and function. Cell. 2004; 116(2):281–297. . 14744438 10.1016/s0092-8674(04)00045-5

[6] Hayes J, Peruzzi PP, Lawler S. MicroRNAs in cancer: biomarkers, functions and therapy. Trends Mol Med. 2014; 20(8):460–469. doi: 10.1016/j.molmed.2014.06.005 . 25027972 10.1016/j.molmed.2014.06.005

[7] Schetter AJ, Leung SY, Sohn JJ, Zanetti KA, Bowman ED, Yanaihara N, et al MicroRNA expression profiles associated with prognosis and therapeutic outcome in colon adenocarcinoma. JAMA. 2008; 299(4):425–436. doi: 10.1001/jama.299.4.425 . 18230780 10.1001/jama.299.4.425 PMC2614237

[8] Dews M, Homayouni A, Yu D, Murphy D, Sevignani C, Wentzel E, et al Augmentation of tumor angiogenesis by a Myc-activated microRNA cluster. Nat Genet. 2006; 38(9):1060–1065. doi: 10.1038/ng1855 . 16878133 10.1038/ng1855 PMC2669546

[9] Yu Y, Nangia-Makker P, Farhana L, G Rajendra S, Levi E, Majumdar AP, et al miR-21 and miR-145 cooperation in regulation of colon cancer stem cells. Mol Cancer. 2015; 14:98 doi: 10.1186/s12943-015-0372-7 . 25928322 10.1186/s12943-015-0372-7 PMC4415383

[10] Madison BB, Jeganathan AN, Mizuno R, Winslow MM, Castells A, Cuatrecasas M, et al Let-7 Represses Carcinogenesis and a Stem Cell Phenotype in the Intestine via Regulation of Hmga2. PLoS Genet. 2015; 11(8):e1005408 doi: 10.1371/journal.pgen.1005408 . 26244988 10.1371/journal.pgen.1005408 PMC4526516

[11] Chen X, Guo X, Zhang H, Xiang Y, Chen J, Yin Y, et al Role of miR-143 targeting KRAS in colorectal tumorigenesis. Oncogene. 2009; 28(10):1385–1392. doi: 10.1038/onc.2008.474 . 19137007 10.1038/onc.2008.474

[12] Landskroner-Eiger S, Moneke I, Sessa WC. miRNAs as modulators of angiogenesis. Cold Spring Harb Perspect Med. 2013; 3(2): a006643 doi: 10.1101/cshperspect.a006643 . 23169571 10.1101/cshperspect.a006643 PMC3552340

[13] Tong JL, Zhang CP, Nie F, Xu XT, Zhu MM, Xiao SD, et al MicroRNA 506 regulates expression of PPAR alpha in hydroxycamptothecin-resistant human colon cancer cells. FEBS Lett. 2011; 585(22):3560–3568. doi: 10.1016/j.febslet.2011.10.021 . 22036718 10.1016/j.febslet.2011.10.021

[14] Gao Y, Xiao Q, Ma H, Li L, Liu J, Feng Y, et al LKB1 inhibits lung cancer progression through lysyl oxidase and extracellular matrix remodeling. Proc Natl Acad Sci U S A. 2010; 107(44):18892–18897. doi: 10.1073/pnas.1004952107 . 20956321 10.1073/pnas.1004952107 PMC2973865

[15] Gale M, Sayegh J, Cao J, Norcia M, Gareiss P, Hoyer D, et al Screen-identified selective inhibitor of lysine demethylase 5A blocks cancer cell growth and drug resistance. Oncotarget. 2016; 7(26):39931–39944. doi: 10.18632/oncotarget.9539 . 27224921 10.18632/oncotarget.9539 PMC5129982

[16] Cao J, Wu L, Zhang SM, Lu M, Cheung WK, Cai W, et al An easy and efficient inducible CRISPR/Cas9 platform with improved specificity for multiple gene targeting. Nucleic Acids Res. 2016; 44(19):e149 doi: 10.1093/nar/gkw660 . 27458201 10.1093/nar/gkw660 PMC5100567

[17] Urbich C, Kaluza D, Frömel T, Knau A, Bennewitz K, Boon RA, et al MicroRNA-27a/b controls endothelial cell repulsion and angiogenesis by targeting semaphorin 6A. Blood. 2012; 119(6):1607–1616. doi: 10.1182/blood-2011-08-373886 . 22184411 10.1182/blood-2011-08-373886

[18] Xu Y, Zhou M, Wang J, Zhao Y, Li S, Zhou B, et al Role of microRNA-27a in down-regulation of angiogenic factor AGGF1 under hypoxia associated with high-grade bladder urothelial carcinoma. Biochim Biophys Acta. 2014; 1842(5):712–725. doi: 10.1016/j.bbadis.2014.01.007 . 24462738 10.1016/j.bbadis.2014.01.007

[19] Park KJ, Cho SB, Park YL, Kim N, Park SY, Myung DS, et al Prospero homeobox 1 mediates the progression of gastric cancer by inducing tumor cell proliferation and lymphangiogenesis. Gastric Cancer. 2017; 20(1):104–115. doi: 10.1007/s10120-015-0592-y . 26759228 10.1007/s10120-015-0592-y

[20] Hong YK, Harvey N, Noh YH, Schacht V, Hirakawa S, Detmar M, et al Prox1 is a master control gene in the program specifying lymphatic endothelial cell fate. Dev Dyn. 2002; 225(3):351–357. doi: 10.1002/dvdy.10163 . 12412020 10.1002/dvdy.10163

[21] Oka M, Iwata C, Suzuki HI, Kiyono K, Morishita Y, Watabe T, et al Inhibition of endogenous TGF-beta signaling enhances lymphangiogenesis. Blood. 2008; 111(9):4571–4579. doi: 10.1182/blood-2007-10-120337 . 18310502 10.1182/blood-2007-10-120337

[22] Karpanen T, Alitalo K. Molecular biology and pathology of lymphangiogenesis. Annu Rev Pathol. 2008; 3: 367–397. doi: 10.1146/annurev.pathmechdis.3.121806.151515 . 18039141 10.1146/annurev.pathmechdis.3.121806.151515

[23] Qiu X, Yao S, Zhang S. Advances in the research on lymphangiogenesis in carcinoma tissues (Review). Oncol Lett. 2010; 1:579–582. doi: 10.3892/ol_00000102 . 22966346 10.3892/ol_00000102 PMC3436362

[24] Fish JE, Santoro MM, Morton SU, Yu S, Yeh RF, Wythe JD, et al miR-126 regulates angiogenic signaling and vascular integrity. Dev Cell. 2008; 15: 272–284. doi: 10.1016/j.devcel.2008.07.008 . 18694566 10.1016/j.devcel.2008.07.008 PMC2604134

[25] Guo C, Sah JF, Beard L, Willson JK, Markowitz SD, Guda K. The noncoding RNA, miR-126, suppresses the growth of neoplastic cells by targeting phosphatidylinositol 3-kinase signaling and is frequently lost in colon cancers. Genes Chromosomes Cancer. 2008; 47: 939–946. doi: 10.1002/gcc.20596 . 18663744 10.1002/gcc.20596 PMC2739997

[26] Nicoli S, Standley C, Walker P, Hurlstone A, Fogarty KE, Lawson ND. MicroRNA-mediated integration of haemodynamics and Vegf signalling during angiogenesis. Nature. 2010; 464: 1196–1200. doi: 10.1038/nature08889 . 20364122 10.1038/nature08889 PMC2914488

[27] Sasahira T, Kurihara M, Bhawal UK, Ueda N, Shimomoto T, Yamamoto K, et al Downregulation of miR-126 induces angiogenesis and lymphangiogenesis by activation of VEGF-A in oral cancer. Br J Cancer. 2012; 107: 700–706. doi: 10.1038/bjc.2012.330 . 22836510 10.1038/bjc.2012.330 PMC3419968

[28] Jones D, Li Y, He Y, Xu Z, Chen H, Min W. Mirtron microRNA-1236 inhibits VEGFR-3 signaling during inflammatory lymphangiogenesis. Arterioscler Thromb Vasc Biol. 2012; 32: 633–642. doi: 10.1161/ATVBAHA.111.243576 . 22223733 10.1161/ATVBAHA.111.243576 PMC3288963

[29] Kazenwadel J, Michael MZ, Harvey NL. Prox1 expression is negatively regulated by miR-181 in endothelial cells. Blood. 2010; 116(13): 2395–2401. doi: 10.1182/blood-2009-12-256297 . 20558617 10.1182/blood-2009-12-256297

[30] Xiao Q, Ge G. Lysyl oxidase, extracellular matrix remodeling and cancer metastasis. Cancer Microenviron. 2012; 5(3):261–273. doi: 10.1007/s12307-012-0105-z . 22528876 10.1007/s12307-012-0105-z PMC3460045

[31] Ferrara N, Gerber HP, LeCouter J. The biology of VEGF and its receptors. Nat Med. 2003; 9(6):669–676. doi: 10.1038/nm0603-669 . 12778165 10.1038/nm0603-669

[32] Xu L, Xiang J, Shen J, Zou X, Zhai S, Yin Y, et al Oncogenic MicroRNA-27a is a Target for Genistein in Ovarian Cancer Cells. Anticancer Agents Med Chem. 2013; 13(7):1126–1132. . 23438830 10.2174/18715206113139990006

[33] Zhao X, Yang L, Hu J. Down-regulation of miR-27a might inhibit proliferation and drug resistance of gastric cancer cells. J Exp Clin Cancer Res. 2011; 30:55 doi: 10.1186/1756-9966-30-55 . 21569481 10.1186/1756-9966-30-55 PMC3120716

[34] Wang Q, Li DC, Li ZF, Liu CX, Xiao YM, Zhang B, et al Upregulation of miR-27a contributes to the malignant transformation of human bronchial epithelial cells induced by SV40 small T antigen. Oncogene. 2011; 30(36):3875–3886. doi: 10.1038/onc.2011.103 . 21460851 10.1038/onc.2011.103

[35] Tang W, Zhu J, Su S, Wu W, Liu Q, Su F, et al MiR-27 as a prognostic marker for breast cancer progression and patient survival. PLoS One. 2012; 7(12):e51702 doi: 10.1371/journal.pone.0051702 . 23240057 10.1371/journal.pone.0051702 PMC3519894

[36] Ma Y, Yu S, Zhao W, Lu Z, Chen J. miR-27a regulates the growth, colony formation and migration of pancreatic cancer cells by targeting Sprouty2. Cancer Lett. 2010; 298(2):150–158. doi: 10.1016/j.canlet.2010.06.012 . 20638779 10.1016/j.canlet.2010.06.012

[37] Venkatesh T, Nagashri MN, Swamy SS, Mohiyuddin SM, Gopinath KS, Kumar A. Primary microcephaly gene MCPH1 shows signatures of tumor suppressors and is regulated by miR-27a in oral squamous cell carcinoma. PLoS One. 2013; 8(3):e54643 doi: 10.1371/journal.pone.0054643 . 23472065 10.1371/journal.pone.0054643 PMC3589425

[38] Tang W, Yu F, Yao H, Cui X, Jiao Y, Lin L, et al miR-27a regulates endothelial differentiation of breast cancer stem like cells. Oncogene. 2014; 33(20):2629–2638. doi: 10.1038/onc.2013.214 . 23752185 10.1038/onc.2013.214

[39] Glover AR, Zhao JT, Gill AJ, Weiss J, Mugridge N, Kim E, et al: MicroRNA-7 as a tumor suppressor and novel therapeutic for adrenocortical carcinoma. Oncotarget. 2015; 6(34):36675–36688. doi: 10.18632/oncotarget.5383 . 26452132 10.18632/oncotarget.5383 PMC4742203

[40] Brognara E, Fabbri E, Montagner G, Gasparello J, Manicardi A, Corradini R, et al: High levels of apoptosis are induced in human glioma cell lines by co-administration of peptide nucleic acids targeting miR‑221 and miR‑222. Int J Oncol. 2016; 48(3):1029–1038. doi: 10.3892/ijo.2015.3308 . 26708164 10.3892/ijo.2015.3308

[41] Miyaki M, Kuroki T. Role of SMAD4 (DPC4) inactivation in human cancer. Biochem Biophys Res Commun. 2003; 306: 799–804. . 12821112 10.1016/s0006-291x(03)01066-0

[42] Shi Y, Massague J. Mechanisms of TGF-beta signaling from cell membrane to the nucleus. Cell. 2003; 113: 685–700. . 12809600 10.1016/s0092-8674(03)00432-x

[43] Huang S, He X, Ding J, Liang L, Zhao Y, Zhang Z, et al Upregulation of miR-23a approximately 27a approximately 24 decreases transforming growth factor-beta-induced tumor-suppressive activities in human hepatocellular carcinoma cells. Int J Cancer. 2008; 123(4):972–978. doi: 10.1002/ijc.23580 . 18508316 10.1002/ijc.23580

[44] Thiagalingam S, Lengauer C, Leach FS, Schutte M, Hahn SA, Overhauser J, et al Evaluation of candidate tumour suppressor genes on chromosome 18 in colon cancers. Nat Genet. 1996; 13: 343–346. doi: 10.1038/ng0796-343 . 8673134 10.1038/ng0796-343

[45] Salovaara R, Roth S, Loukola A, Launonen V, Sistonen P, Avizienyte E, et al Frequent loss of SMAD4/DPC4 protein in colon cancers. Gut. 2002; 51: 56–59. . 12077092 10.1136/gut.51.1.56 PMC1773263

[46] Yang J, Wang Y, Zeng Z, Qiao L, Zhuang L, Gao Q, et al Smad4 deletion in blood vessel endothelial cells promotes ovarian cancer metastasis. Int J Oncol. 2017; 50(5):1693–1700. doi: 10.3892/ijo.2017.3957 . 28393199 10.3892/ijo.2017.3957

[47] Li X, Liu B, Xiao J, Yuan Y, Ma J, Zhang Y. Roles of VEGF-C and Smad4 in the lymphangiogenesis, lymphatic metastasis, and prognosis in colon cancer. J Gastrointest Surg. 2011;15(11): 2001–2010. doi: 10.1007/s11605-011-1627-2 . 21786062 10.1007/s11605-011-1627-2

[48] Wan X, Huang W, Yang S, Zhang Y, Zhang P, Kong Z, et al Androgen-induced miR-27A acted as a tumor suppressor by targeting MAP2K4 and mediated prostate cancer progression. Int J Biochem Cell Biol. 2016; 79:249–260. doi: 10.1016/j.biocel.2016.08.043 . 27594411 10.1016/j.biocel.2016.08.043

[49] Hiwatashi K, Ueno S, Sakoda M, Kubo F, Tateno T, Kurahara H, et al Strong Smad4 expression correlates with poor prognosis after surgery in patients with hepatocellular carcinoma. Ann Surg Oncol. 2009; 16(11):3176–82. doi: 10.1245/s10434-009-0614-2 . 19626374 10.1245/s10434-009-0614-2

